# The competency index for clinical research professionals: a potential tool for competency-based clinical research academic program evaluation

**DOI:** 10.3389/fmed.2024.1291667

**Published:** 2024-03-26

**Authors:** Carolynn Thomas Jones, Xin Liu, Carlton A. Hornung, Jessica Fritter, Marjorie V. Neidecker

**Affiliations:** ^1^Center for Clinical Translational Science, College of Nursing, The Ohio State University, Columbus, OH, United States; ^2^Division of Biostatistics, College of Public Health, The Ohio State University, Columbus, OH, United States; ^3^Department of Medicine, University of Louisville, Louisville, KY, United States; ^4^College of Pharmacy, The Ohio State University, Columbus, OH, United States

**Keywords:** clinical trial competency, program evaluation, competency-based education, academic program in clinical research, clinical research professionals

## Abstract

**Background:**

Accreditation of graduate academic programs in clinical research requires demonstration of program achievement of Joint Task Force for Clinical Trial Competence-based standards. Evaluation of graduate programs include enrollment, student grades, skills-based outcomes, and completion rates, in addition to other measures. Standardized measures of competence would be useful.

**Methods:**

We used the Competency Index for Clinical Research Professionals (CICRP), in a separate-sample pretest-posttest study to measure self-confidence or self-efficacy in clinical research competency comparing cohorts of students entering and completing a master’s degree program in clinical research across three semesters (summer 2021 – spring 2022). CICRP is a 20-item Likert scale questionnaire (0 = Not at all confident; 10 = extremely confident).

**Results:**

The study sample of 110 students (54 in the entry course, 56 in the exit course) showed overall 80.9% entered the program with only a baccalaureate degree and 55.5% had no prior experience in managing clinical trial research. Cronbach alpha for the instrument showed a high level of content validity (range 0.93–0.98). Median CICRP item rating range at entry was [1, 6] and at exit [7, 10]. Mean CICRP total score (sum of 20 items) at entry was 72.7 (SD 41.9) vs. 167.0 (SD 21.1) at exit (*p* < 0.001). Mean total score at program entry increased with increasing years of clinical trial management experience but attenuated at program exit.

**Conclusion:**

This is the first use of the CICRP for academic program evaluation. The CICRP may be a useful tool for competency-based academic program evaluation, in addition to other measures of program excellence.

## 1 Introduction

Academic programs in clinical research have evolved over the past two decades to provide an educational pathway for clinical research professionals for chosen career paths in clinical research. Academic programs may range from associate degrees, undergraduate or graduate certificates, undergraduate degrees and master’s degrees in clinical research management and regulatory affairs. Many of these programs are distance-based and asynchronous, enrolling students nationally and internationally. Other graduate programs also support more advanced clinical translational research and regulatory science education for doctorally prepared clinical translational scientists (e.g., physicians, pharmacologists, and basic scientists).

**Figure 1:**
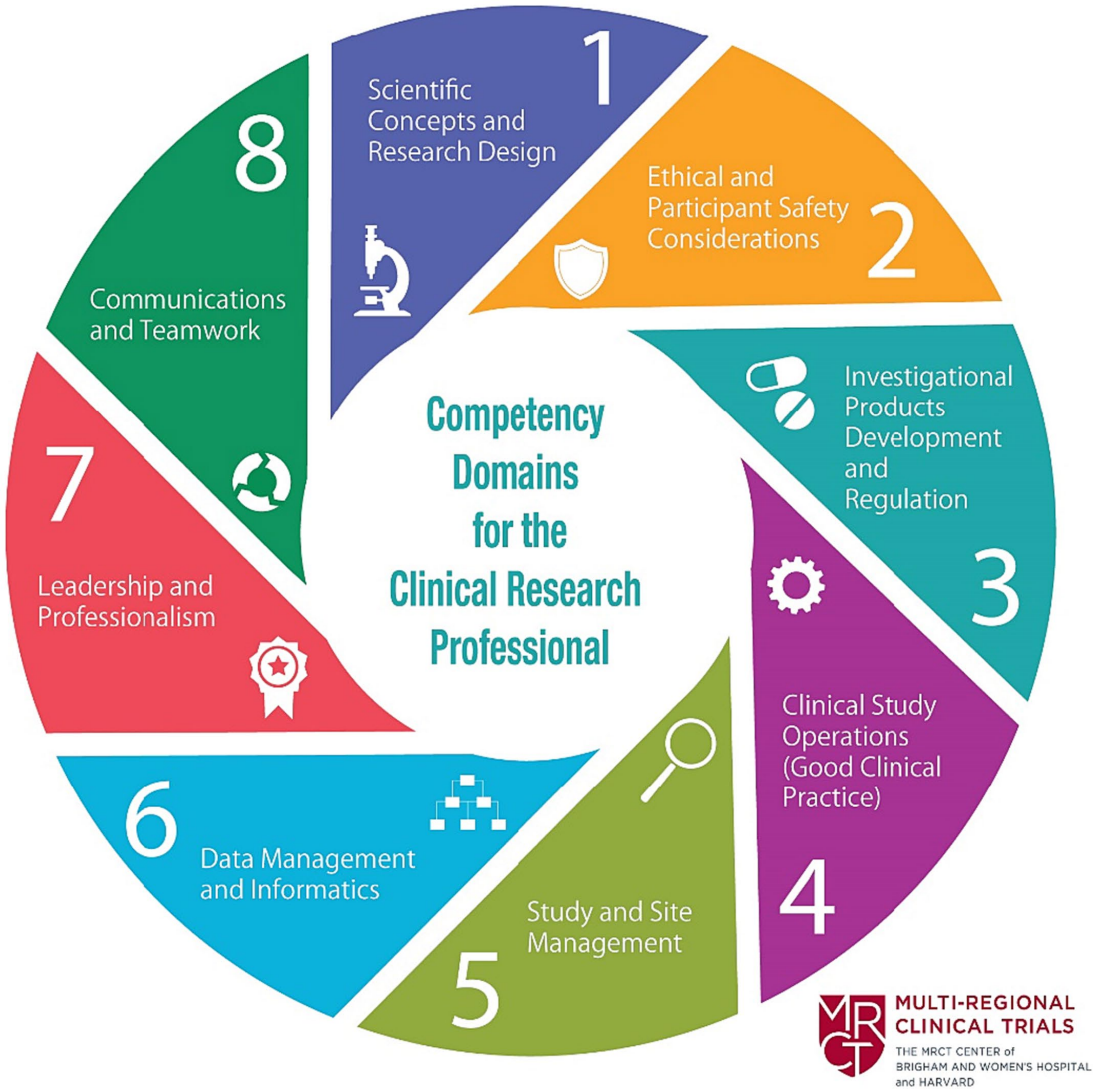
Joint task force clinical trial competency framework (3). Reprinted with permission from Barbara Bierer, Director, Multiregional Clinical Trials Center (MRCT) at https://mrctcenter.org/clinical-trial-competency/. Copyright 2024 Brigham and Women’s Hospital Division of Global Health Equity.

The Joint Task Force (JTF) for Clinical Trial Competency (JTF Framework) is an international team of investigators, educators, sponsors and clinical research professionals that has developed a framework that defines the knowledge, skills and attitudes necessary for conducting safe, ethical, and high-quality clinical research. This group published core competencies in clinical research, harmonizing evolving work in role-based competencies at the time (1, 2) (Figure 1). Subsequent research on the JTF Framework included a global survey applied to competency relevance to roles and training needs in clinical trials (3). Since that time, the JTF Framework has been updated to include illustrated leveling and project management. The JTF website is maintained by the Multi-Regional Clinical Trials Center at Harvard University (4–6).

In 2018, a factor analysis of the global survey data for non-investigator, clinical research professionals working in the United States and Canada resulted in a short-form 20-item competency index assessment tool called the Competency Index for Clinical Research Professionals (CICRP) (Table 1) that used a 0–10 Likert scale (7). The tool analysis included five empirical domain subscales: I. General Operation and Management of Clinical Trials, II. Medicines Development, III. Ethics and Participant Safety, IV. Data Collection and Management, and V. Scientific Concepts in Clinical Research (CICRP-I). The scale was used in a subsequent study exploring the use of the index to compare self-perceived self-efficacy in performing clinical trial skills among clinical research professionals (CRPs) working at academic medical center settings, other site settings and students of academic programs in clinical research. This study assessed the importance of clinical trial experience and academic education in CRPs (8). This index, known as CICRP-II, measured routine functions and advanced functions of clinical research professionals (8).

**Table 1:** Competency Index for Clinical Research Professionals (CICRP) criteria.

CICRP items	JTF competency domain(s)*	CICRP empirical domain(s)**
Describe the role and process for monitoring a study.	(4)	I, III
Describe the roles and responsibilities of various institutions participating in the medicines development process.	(3)	II
Compare and contrast clinical care and clinical management of research participants.	(2)	I, III, V
Summarize the process of electronic data capture (EDC) and the importance of information technology in data collection, capture and management.	(6)	IV
Explain the elements (statistical, epidemiological, and operational) of clinical and translational study design.	(1)	V
Identify the legal responsibilities, issues, liabilities, and accountability that are involved in the conduct of a clinical trial.	(5)	I
Explain the medicines development process and the activities, which integrate commercial realities into the life cycle management of medical products.	(3)	II
Compare the requirements for human subject protection and privacy under different national and international regulation and ensures their implementation throughout all phases of a clinical study.	(2), (4)	III
Describe the significance of data quality assurance systems and how SOPs are used to guide these processes.	(6)	I, IV
Critically analyze study results with an understanding of therapeutic and comparative effectiveness.	(1)	V
Summarize the legislative and regulatory framework, which supports the development and registration of medicines, devices and biologicals and ensures their safety, efficacy and quality.	(3)	II
Describe the ethical issues involved when dealing with vulnerable populations and the need for additional safeguards.	(2)	III
Compare and contrast the regulations and guidelines of global regulatory bodies relating to the conduct of clinical trials.	(4)	I, V
Describe the specific processes and phases that must be followed for the regulatory authority to approve the marketing authorization for a medical product.	(3)	II
Differentiate the types of adverse events which occur during clinical trials, understand the identification process for AEs and describe the reporting requirements to IRBs/IECs, sponsors and regulatory authorities.	(4)	II, III
Describe the reporting requirements of global regulatory bodies relating to clinical trial conduct.	(4)	I, IV, V
Describe the impact of cultural diversity and the need for cultural competence in the design and conduct of clinical research.	(7)	IV
Define the concepts of "clinical equipoise" and "therapeutic misconception" as they relate to the conduct of a clinical trial.	(2)	I
Apply management concepts and effective training methods to manage risk and improve quality in the conduct of a clinical research study.	(5)	I
Identify and apply the professional guidelines and codes of ethics, which apply to the conduct of clinical research.	(7)	I, IV

*JTF competency domains	**CICRP empirical domains
(1) Scientific concepts and research design (2) Ethical and participant safety considerations (3) Investigational products development and regulation (4) Clinical study operations (good clinical practice) (5) Study and site management (6) Data management and informatics (7) Leadership and professionalism (8) Communications and teamwork	General operation and management of clinical trials Medicines development Ethics and participant safety Data collection and management Scientific concepts in clinical research

CICRP, Competency Index for Clinical Research Professionals; JTF, Joint Task Force; SOP, Standard Operating Procedures; AEs, adverse events; IRBs, Institutional Review Boards; IECs, Independent Ethics Committees.

The Consortium of Academic Programs in Clinical Research, established an accreditation pathway for academic programs in clinical research. Accreditation is offered by Commission on Accreditation of Allied Health Education Programs (CAAHEP) and is administered by the Committee on Accreditation of Academic Programs in Clinical Research (CAAPCR) (9). The CAAPCR accreditation standards incorporate the JTF Competency Framework for competency-based clinical research educational programs. The self-study process requires gathering numerous student, course, program and institutional evaluation materials and data to address the specific requirements for the CAAPCR standards and guidelines. Program evaluation measures include enrollment; retention and graduation metrics; and student and course demonstration of achieving clinical research competencies by analysis of competency-based course assignments mapped to program goals, course objectives and the JTF Framework.

The authors are reporting on the use of the 20-item CICRP instrument as an evaluation tool in a 100% online asynchronous master’s degree program in clinical research (Master of Clinical Research, MCR) with specializations in both clinical research management and regulatory affairs at a midwestern public institution in the United States, with a major academic medical center. Students complete 12 graduate courses (36 credit hours total) consisting of seven core courses, four specialization courses and a culminating project course. Students are accepted into the program three times per year (spring, summer, and autumn) using a holistic admissions method, including required undergraduate GPA of 3.0. Prior clinical research experience is not a pre-requisite to admission. Courses are delivered using a well-established learning management system adopted by the university and taught by faculty with experience in clinical research, clinical trials, pharmacology, bioethics, and biostatistics. The program curriculum is mapped to the JTF Framework with a heavy distribution of JTF competencies across the core courses and more focused JTF competencies across the specialization courses. The final course allows students to select one of five culminating project options: develop an integrative review, develop a research protocol/proposal, develop a manuscript on a clinical research topic, develop and perform a clinical research-related project, or work with a mentor in a focused research opportunity. Another deliverable in the culminating project course is the development of an ePortfolio that included evidence of acquired JTF competency skillsets and an essay on each of the JTF competency domains reflecting on their learning in each domain and future learning and experiential goals as a clinical research professional. We included applied real-world assignments to provide authentic learning for students to enhance the competency-based nature of our asynchronous learning environment. Table 2 provides examples from a subset of applied competency-based assignments found in courses in the curriculum. Furthermore, our courses were structured using program-designed, learner-centric module templates, applying collaborative learning pedagogy including forming a course community, providing opportunities for interactive discussion, and requiring ongoing teacher scaffolding through frequent input. This pedagogy is in keeping with the best practices for online collaborative education (10). The program requires that students maintain a B- or above final grade in all completed courses and an overall GPA of 3.0 to graduate.

**Table 2:** Subset of authentic applied assignments in the master’s program core courses aligned to JTF competency domai.

Applied assignment	JTF competency domain(s)
Develop an IND submission for an assigned study	(3) Investigational products development and regulation
Describe and analyze a manuscript’s statistical methods, results for an assigned study and dataset	(1) Scientific concepts and research design
Develop a PICOT question and research proposal.	(1) Scientific concepts and research design
Develop a quality management plan for a clinical research site and study.	(5) Study and site management
Demonstrate the correct use of electronic case report form system from perspective of the sponsor, monitor and coordinator.	(6) Data management and informatics
Analyze and discuss bioethical case studies applying regulations.	(2) Ethical and participant safety considerations
Develop an IRB submission and informed consent form for an assigned clinical study.	(2) Ethical and participant safety (4) Clinical study operations (GCPs)
Develop a recruitment analysis and plan for an assigned clinical trial.	(2) Ethical and participant safety (4) Clinical study operations
Work as a team to develop a data management plan for an assigned study.	(6) Data management and informatics (8) Communication and teamwork
Conduct and present a risk analysis of a planned study.	(5) Study and site management (4) Clinical study operations (GCPs)
Generate a CAPA and SOPs based on findings from FDA warning letters.	(4) Clinical study operations (GCPs) (3) Investigational products development and regulation
Create case studies and scripts demonstrating the application of crucial conversations principles in a conflict between parties occurring at a clinical research site.	(7) Leadership and professionalism (8) Communication and teamwork

CAPA, Corrective and Preventive Action; JTF, Joint Task Force; SOP, Standard Operating Procedures; FDA, Food and Drug Administration; IND, Investigational New Drug; IRB, Institutional Review Board; GCPs, Good Clinical Practices; PICOT, population/patient, intervention, comparison, outcome, and time.

While the master’s program evaluated competence for clinical research professional roles through students’ assignments, ePortfolios and culminating projects, a standardized assessment tool was lacking. The program aimed to supplement the existing measures of competency by including the CICRP questionnaire as a program evaluation tool. The purpose of this study is to describe the results of entry versus exit course assessments using the CICRP tool for academic competency-based program evaluation.

## 2 Methods

### 2.1 CICRP survey instrument

**Figure 2:**
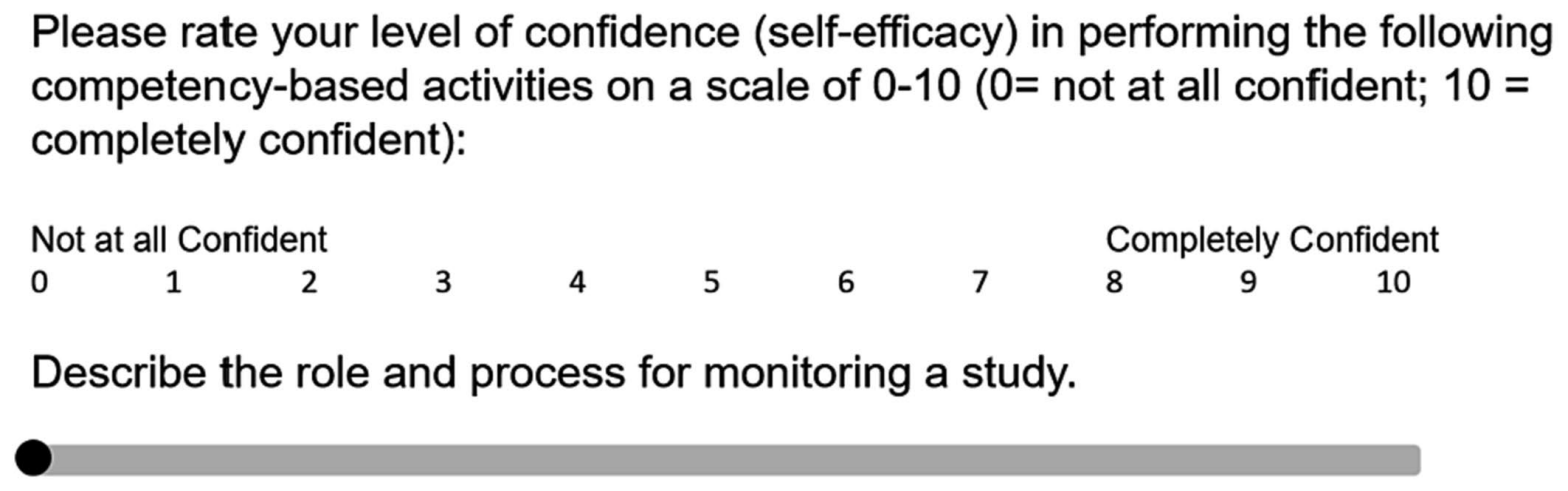
Illustration of CICRP question in Qualtrics^XM^ using a slider scale. CICRP, Competency Index for Clinical Research Professionals.

For the purpose of using the CICRP instrument as a program evaluation tool, we used composite scores from the 20-item scale without the CICRP I or II subscale analyses (6, 11). Using a separate-sample pre-post study design, we administered the CICRP questionnaire to students in the entry and exit courses of our clinical research master’s degree program during the 2021–2022 academic year. We created a Qualtrics^XM^ (Provo, Utah) survey instrument including the 20 CICRP items asking students to rate their self-efficacy in performing each item (Table 1) using a Likert slider scale from 0 to 10 (0 = not at all confident; 10 = extremely confident) (Figure 2). The survey required a response to each item, and if the respondent intended to select zero, a click or tap on zero was required. Students could participate in the survey on a desktop or a mobile device. We posted links to the survey in the learning management system in course modules and on the course calendar. We also sent reminders to take the CICRP survey through course announcements that generated a notice to their student E-mail inbox.

We included the CICRP Qualtrics^XM^ survey as a required non-graded assessment in the beginning of the students’ initial program course (course number MCR 7770) and again at the end of their final program course (course number MCR 7599). We designed this study to measure and compare entering and exiting students to assess whether the CICRP tool had utility for program evaluation. Prior to commencing the survey, students were provided with an informed consent for this study with a prompt to proceed if they consented. The survey study was granted exempt approval by the institution’s Institutional Review Board.

### 2.2 Statistical methods

We describe the two student groups entering and exiting the program by highest degree at program entry, years of clinical research experience, whether being a nurse, and whether holding clinical research certification (12, 13). The Qualtrics^XM^ data set includes the CICRP ratings of the students from the entry course in semesters Summer 2021 (SU21) and Autumn 2021 (AU21) (the entry course is not offered during spring semester). In the exit course, the CICRP survey was conducted with students toward the end of the course during SU21, AU 21 and Spring 2022 (SP22). For each of the 20 survey items we used the Likert scale based on CICRP from zero to 10 (7). *T*he summation of the combined 20 CICRP items is a good tool to evaluate clinical trial core competencies overall (11), which we denote as the CICRP total score *(range 0–200).*

Because each CICRP item contains 11 categories (0, 1,…,10) it would be reasonable to treat the rating scale as continuous interval data (14, 15). For each item, a higher rating means greater perceived efficacy for that item, *while* a higher CICRP total score *signifies greater perceived competency*.

CICRP total scores were first directly compared between the students taking the program entry course and the program exit course. Students put their student email ID in the tool to ensure no duplicate entries. When the dataset was downloaded, those identifiers were removed before analysis to preserve anonymity. We used linear regression to adjust for these potential confounders such as “semester” and “highest degree at program entry.

## 3 Results

### 3.1 Participant characteristics

All students were required to complete the CICRP survey as a non-graded assignment. The number of students in the entry course taking the CICRP was 23 in SU21 and 31 in AU21 (total *n* = 54). The number of students taking the CICRP in the exit course was 27 in SU21, 14 in AU21 and 15 in SP22 (total *n* = 56) (Table 3).

**Table 3:** Participant education, experience, nursing and certification.

	Entry course		Exit course			Entry course total	Exit course total	Overall	*p-*value

	SU21	AU21	SU21	AU21	SP22				
	*n* = 23	*n* = 31	*n* = 27	*n* = 14	*n* = 15	*n* = 54	*n* = 56	*n* = 110	
Highest level of education completed before entering master's program									
Bachelor's degree	95.7%	93.5%	63.0%	71.4%	73.3%	94.4%	67.9%	80.9%	<0.001
Master's degree	0.0%	3.2%	25.9%	28.6%	13.3%	1.9%	23.2%	12.7%	
Doctorate degree	4.3%	3.2%	11.1%	0.0%	13.3%	3.7%	8.9%	6.4%	
Years of experience managing clinical trials research									
None	65.2%	54.8%	48.1%	35.7%	73.3%	59.3%	51.8%	55.5%	0.344
< 1 year	13.0%	12.9%	3.7%	7.1%	6.7%	13.0%	5.4%	9.1%	
1–2 years	4.3%	0.0%	7.4%	14.3%	6.7%	1.9%	8.9%	5.5%	
>2–3 years	13.0%	12.9%	18.5%	21.4%	0.0%	13.0%	14.3%	13.6%	
>3–5 Years	0.0%	6.5%	14.8%	7.1%	6.7%	3.7%	10.7%	7.3%	
>5–10 years	4.3%	9.7%	3.7%	7.1%	6.7%	7.4%	5.4%	6.4%	
>10–20 years	0.0%	3.2%	3.7%	7.1%	0.0%	1.9%	3.6%	2.7%	
Nurse									
	4.3%	29.0%	14.8%	7.1%	13.3%	18.5%	12.5%	15.5%	0.437
Clinical research certification									
	0.0%	16.1%	0.0%	14.3%	6.7%	9.3%	5.4%	7.3%	0.485

SU21, Summer 2021 semester; AU21, Autumn 2021 semester; SP22, Spring 2022 semester; n, number.

As required in the program, all students had a bachelor’s degree; however, in the exit course, a greater proportion of students had entered the program already holding a graduate degree (5.6% in the entry course vs. 32.1% in the exit course, *p* < 0.001). We asked students, “What is your current level of experience in clinical research?” Of the students taking the CICRP in the entry course, 59.3% indicated they had no experience, while 25.9% had more than 2 years of experience. Among students taking the CICRP in their exit course, responses showed more *but statistically* insignificant levels of experience: 51.8% indicated no prior experience, while 33.9% indicated more than 2 years (*p* = 0.344). The program aims to increase its enrollment of nurses to the program so the question, “Are you a nurse” provides data for correlational scores in future analyses.

### 3.2 CICRP total scores

Our analysis explored the question, **“**Does the Master of Clinical Research program have any significant effect on the improvement of students’ self-efficacy in clinical trial core competences in terms of the CICRP ratings.” We calculated Cronbach’s alpha (16) for each assessment with ratings ranging from 0.93 to 0.98 (Table 4) showing a high degree of content and face validity. The range for combined entry course median item ratings were 0–6, and the range for exit course median item ratings were 7–10.

**Table 4:** CICRP median item ratings and mean total score by semester and course.

CICRP items	Entry course		Exit course		
	SU21	AU21	SU21	AU21	SP22
Describe the role and process for monitoring a study.	4	3	9	8	7
Describe the roles and responsibilities of various institutions participating in the medicines development process.	3	3	8	9.5	7
Compare and contrast clinical care and clinical management of research participants.	4	5	9	9.5	8
Summarize the process of electronic data capture (EDC) and the importance of information technology in data collection, capture and management.	4	3	9	10	7
Explain the elements (statistical, epidemiological and operational) of clinical and translational study design.	4	2	8	8	7
Identify the legal responsibilities, issues, liabilities and accountability that are involved in the conduct of a clinical trial.	3	3	8	9	8
Explain the medicines development process and the activities, which integrate commercial realities into the life cycle management of medical products.	3	2	8	9	8
Compare the requirements for human subject protection and privacy under different national and international regulation and ensures their implementation throughout all phases of a clinical study.	5	4	9	10	7
Describe the significance of data quality assurance systems and how SOPs are used to guide these processes.	4	5	9	10	7
Critically analyze study results with an understanding of therapeutic and comparative effectiveness.	4	4	8	9	8
Summarize the legislative and regulatory framework, which supports the development and registration of medicines, devices and biologicals and ensures their safety, efficacy and quality.	3	2	8	9	7
Describe the ethical issues involved when dealing with vulnerable populations and the need for additional safeguards.	6	6	9	10	8
Compare and contrast the regulations and guidelines of global regulatory bodies relating to the conduct of clinical trials.	3	2	8	9.5	7
Describe the specific processes and phases, which must be followed in order for the regulatory authority to approve the marketing authorization for a medical product.	3	2	8	9	8
Differentiate the different types of adverse events which occur during clinical trials, understand the identification process for AEs and describe the reporting requirements to IRBs/IECs, sponsors and regulatory authorities.	3	3	9	10	8
Describe the reporting requirements of global regulatory bodies relating to clinical trial conduct.	3	2	8	8.5	7
Describe the impact of cultural diversity and the need for cultural competence in the design and conduct of clinical research.	4	5	9	9.5	9
Define the concepts of "clinical equipoise" and "therapeutic misconception" as they relate to the conduct of a clinical trial.	3	1	8	9	8
Apply management concepts and effective training methods to manage risk and improve quality in the conduct of a clinical research study.	3	3	9	9.5	8
Identify and apply the professional guidelines and codes of ethics, which apply to the conduct of clinical research.	4	4	9	10	8
Cronbach’s alpha	0.96	0.98	0.93	0.98	0.94
CICRP total score					
Course semester mean (standard deviation)	72.1 (33.6)	73.2 (47.7)	168.9 (16.7)	171.1 (29.7)	159.8 (18.3)
Course overall mean (standard deviation)*	72.7 (41.9)		167.0 (21.1)		

*p < 0.001. SU21, Summer 2021 semester; AU21, Autumn 2021 semester; SP22, Spring 2022 semester; CICRP, Competency Index for Clinical Research Professionals; SOP, Standard Operating Procedures; IRBs/IECs, Institutional Review Boards/Independent Ethics Committees.

We conducted parametric and non-parametric two-sample tests to see whether the group of individuals leaving the program have significantly higher mean CICRP total scores compared to the group of individuals entering the program, 167.0 (SD 21.1) vs. 72.7 (SD 41.9), respectfully. Both the Welch’s two-sample t-test and Wilcoxon rank-sum test show very significant differences between the group of students entering the program and leaving the program (*p* < 0.001).

**Figure 3:**
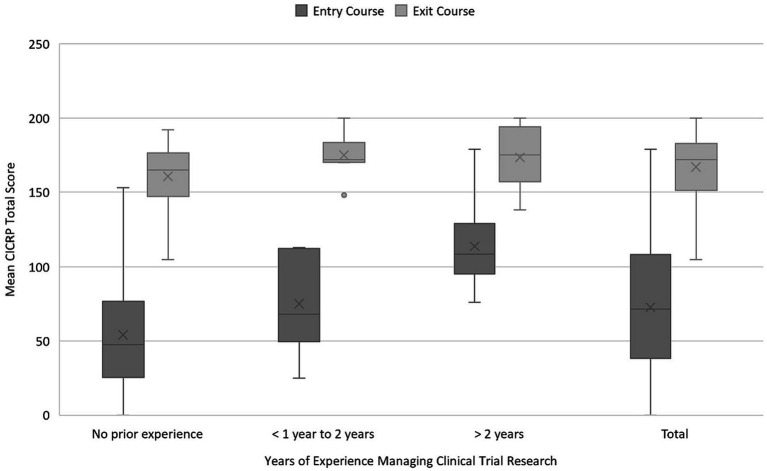
Mean CICRP total score by years of experience. CICRP, Competency Index for Clinical Research Professionals.

Correlations between years of experience and median total scores of each group were difficult to accurately calculate because of the large percentage of students who had no or < 1 year of clinical research experience at the time of the survey. Those in other experience categories were too few to draw meaningful conclusions. However, when combining years of experience into three categories, a significant increase in mean CICRP total score is seen at each experience level between program entry and program exit: no prior experience 54.1 (SD 35.9) vs. 160.7 (SD 21.7), <1 to 2 years 75.2 (SD 33.5) vs. 174.9 (SD 14.8), >2 years 113.9 (SD 28.4) vs. 173.4 (SD 20.3) (*p* < 0.001) (Figure 3).

We further implemented a linear regression of CICRP total scores by course, semester, highest degree at program entry, years of experience, whether being a nurse and clinical research certification to see the effect of course adjusting for other available covariates. The linear regression has a result that, adjusting for available covariates, individuals taking the exit course have a mean CICRP total score 92.690 (*p* < 0.001) higher than individuals taking the entry course. The diagnostic plot of the linear regression does not show signs of fundamental deviation from a normal distribution and generalized variance-inflation factors do not show signs of collinearity. There are significant differences in the variances of different course and semester groups based on Levene’s test. Therefore, we used a general linear model (17) allowing different variances for different course and semester groups. The general linear model does produce a better fit in terms of diagnostic plot, but the result is very close to the ordinary linear model with course coefficient 94.750 (*p* < 0.001). We also carried out backward selection of variables based on the change of courses’ coefficient and *p*-values to omit unnecessary adjustment and to improve precision for estimate of courses’ coefficient. Though we did not find any noticeable changes in the estimates of the course coefficient. The CICRP total scores for these data demonstrate relatively consistent results for students entering and completing the master’s program.

## 4 Discussion

As clinical research competency-based educational programs prepare for accreditation, having a standardized competency evaluation measure such as the CICRP could be a useful program evaluation tool. Competency indexes have been used to evaluate clinical research trainees and educational programs in translational research. The Clinical Research Appraisal Inventory (CRAI) was a 92-item set of competencies for clinical and translational investigators. Robinson et al. created and evaluated a 12-item abbreviated CRAI instrument that was used to evaluate investigator trainees and their acquisition of perceived competence in clinical research (18, 19). Our study presents a potential program evaluation tool for usefulness in assessing whether our competency-based academic program is meeting the JTF Competency needs of students targeting clinical research professional roles. The assessment tool had high Cronbach’s alpha demonstrating a high level of internal consistency. Moreover, these data from our program demonstrate acquisition of competence in the areas of scientific concepts and research design and investigational product development, areas that have been shown to be deficits in the field (20).

A limitation of our study is that it did not measure a head-to-head (entry and exit) pre-test and post-test total scores matched to individual students. Rather, we compare entering students as a cohort (those taking entry course) to graduating students (those taking final course) as an initial pilot to determine feasibility of the index for program evaluation. Furthermore, we found that the graduating cohort in our study appeared to have greater levels of clinical research experience than those entering the program. This may be partially because students in our cohort gained employment in clinical research during their tenure as a student. The graduate students enrolled in our professional master’s degree vary in their progression through the program. Some may take one to two courses per semester (part-time) or three to four courses per semester (full-time). Moreover, some students take semesters off for professional or personal reasons and return at varying time-points, especially during the COVID-19 pandemic. Ideally, we would have assessed individual students and compare total scores at program entry and exit; however, for feasibility purposes we initially wanted to evaluate the tool for usefulness in program evaluation. Future assessments should match specific individual student pre- and post- CICRP total scores and conduct more in-depth assessments of correlations. Another limitation of this study is that it is applicable to students in a specific United States (U.S.) master’s degree program and may not be applicable to students in other U.S. programs or students internationally.

## 5 Conclusion

The Competency Index for Clinical Research Professionals (CICRP) is a short form (20-item) competency index for the JTF Clinical Trial Competencies. It is a useful tool to measure self-efficacy in clinical trial skillsets for clinical research professionals. Used as a pre-test and post-test for students entering and graduating from a graduate-level clinical research academic program, the tool may contribute to evaluate effectiveness of the program, in addition to other program evaluation criteria such as course deliverables, student e-Portfolios, grade point average (GPA), completion rates and successful employment as clinical research professionals. Future research on the use of the tool in program evaluation is warranted.

## Data availability statement

The raw data supporting the conclusions of this article will be made available by the authors, without undue reservation.

## Ethics statement

The studies involving humans were approved by the Ohio State University IRB Columbus, United States. The studies were conducted in accordance with the local legislation and institutional requirements. The participants provided their written informed consent to participate in this study.

## Author contributions

CJ: Conceptualization, Data curation, Formal analysis, Funding acquisition, Investigation, Methodology, Project administration, Resources, Supervision, Writing – original draft, Writing – review & editing, Software, Validation, Visualization. XL: Data curation, Formal analysis, Investigation, Methodology, Validation, Writing – original draft, Writing – review & editing, Software, Visualization. CH: Investigation, Validation, Writing – original draft, Writing – review & editing, Methodology. JF: Data curation, Investigation, Project administration, Validation, Writing – original draft, Writing – review & editing. MN: Conceptualization, Data curation, Formal analysis, Investigation, Methodology, Software, Validation, Visualization, Writing – original draft, Writing – review & editing.
